# Optimization of the Composition of Mineral–Asphalt Mixture for Pothole Repairs Based on Reclaimed Asphalt Using Innovative Chemical Activators

**DOI:** 10.3390/ma18122848

**Published:** 2025-06-17

**Authors:** Marcin Gajewski, Dzmitry Busel, Wojciech Bańkowski, Renata Horodecka, Aleksander Butko

**Affiliations:** 1 Road and Bridge Research Institute, 03-302 Warsaw, Poland; marcin.gajewski@ibdim.edu.pl (M.G.); renata.horodecka@ibdim.edu.pl (R.H.); 2 Glabus Sp. Zoo, 00-682 Warsaw, Poland; termopm@gmail.com; 3 UAB Toro Prekyba, LT-07133 Vilnius, Lithuania; info@torotr.eu

**Keywords:** RAP, recycling, pothole repair mix, asphalt mixes, chemical activator, patch repair, reclaimed asphalt

## Abstract

This paper proposes two chemical activators that are used to prepare mixtures for pothole repairs based on reclaimed asphalt. Repair mixtures, especially those used as “cold” mixes, must be characterized by special properties because they are primarily used in the winter period, i.e., at low temperatures and high humidity. The proposed additives W1 and W2 affect the functional and rheological properties of asphalts. Therefore, their influence on the behavior of asphalt 35/50 in three states, i.e., non-aged, after technological aging, and after service aging, was examined. At the same time, the issue of determining the optimal mass content of additives in relation to the aged binder was undertaken using the original methodology applying the MSCR test. In the latter part of the paper, the influence of additives W1 and W2 on asphalt recovered from reclaimed asphalt (aged in real conditions) was also analyzed and asphalt mixtures for pothole repairs were made. For these mixtures, the average contact stresses and tensile strength were determined as a function of time and conditioning temperature, giving positive results in terms of practical application of the proposed solution. The obtained mixtures were successfully used to fill potholes in winter weather conditions.

## 1. Introduction

The main objective of the article is to present a new type of asphalt mixture for cold pothole repairs consisting of reclaimed asphalt (RAP) activated with an original chemical additive. The optimization process in the selection of the variant and amount of the additive and the study of the strength properties of asphalt mixtures in different composition variants are investigated. The presented solution concerns two important issues related to recycling and maintenance of the pavement. In these areas, the innovativeness of the solution can be indicated. The first is related to the issues of recycling through the use of RAP as the basic component of the repair mixture. Currently used additives in this type of solution are based mainly on new aggregates and new binders/adhesives, which leads to the need to use non-renewable natural resources. In this case, it is planned that RAP will be the basic component, and in special cases, only a small amount of new material in the form of fine and coarse aggregate will be used. The aspect of recycling is directly in line with the principles of the closed-loop economy and allows for the reduction of environmental costs [1,2].

The second aspect will be the use of an innovative binder that will replace currently used agents. The technological solution with this additive will be universal for use—independent of temperature/atmospheric conditions. Currently, asphalt emulsions are used in the summer, and in the winter, binders based on oils or other liquefying substances, which are often environmentally sensitive.

Issues related to the preparation and analysis of mixtures for current pothole repairs have been developed in the literature for years [3,4]. Similarly to the case of mixtures for the construction of new asphalt pavements, two basic technologies are considered, i.e., the “cold” mix technology and the “hot” mix technology. However, it turns out that mixtures for repairs are usually used in such conditions that the use of the “hot” technology is ineffective and sometimes even impossible due to weather conditions. Therefore, mainly solutions in the “cold” technology are sought [5] with an additional requirement resulting mainly from the fact that repairs are carried out in conditions of increased humidity (rainfall, snowfall, lack of possibility to dry the pothole), i.e., the bonding material should not be sensitive to increased humidity [6,7]. This leads to the use of non-standard binders such as epoxy materials [8,9,10], water–asphalt emulsions, fly ash, chemical agents (e.g., PVAC, etc.), bio-additives [11], or reinforcing fibers [4].

For example, in the papers [12,13] the suitability of the mixture for pothole repairs in the region with low temperatures and significant snowfall was verified. The analysis took into account such elements as the quality of interface surface preparation, the type of compaction equipment used, or weather conditions at the time of repair. The conclusions from the statistically analyzed results were clearly negative in the case of the technologies used in Japan in the years 2000–2010. In [14] attention was drawn to another very important element influencing the correctness and durability of pothole repairs using a cold mix, i.e., the properties of the interface between the repair material and the damaged surface layer. The necessity of proper surface preparation was indicated, as this has a very significant impact on the durability of the repair. In the aforementioned publication, among the factors influencing the quality of the interface between materials, the influence of temperature at which the repair was carried out, humidity, type of repair material, and type of material applied on the surface in order to increase the degree of adhesion or evenness of the surface after the repair was examined. It turned out that the most important factor influencing the quality of the bond between materials was the type of surfactant improving the adhesion properties in the interface. The subject of [15] is similar, in which the effectiveness of repair materials with asphalt and polymer binder is assessed in the context of the ambient temperature at the time of its application.

The use of cold mixes for pothole repairs requires the use of non-standard technologies [16] and non-standard experimental tests [17,18]. At the initial stage of designing a cold repair mix, the reference parameter and assessment of the mixes is the indirect tensile strength (ITS) [19,20,21]. However, other methods proposed in [17] based on the so-called Marshall sample indentation with and without lateral confinement are rather insufficient; see also [22,23]. In practice, the indentation test is a test of resistance to cracking and separation of asphalt mixtures, and its advantage is the simplicity of execution, e.g., in comparison to the typical SCB test [15,24]. Among the non-standard methods, the MSCR test used to assess the optimal value of the mass content of the chemical activator in relation to the aged asphalt binder based on the author’s procedure should be mentioned.

When analyzing the possibility of reusing reclaimed asphalt, it is necessary to discuss the aging processes occurring in asphalt pavements [25,26]. Considering the change in the properties of asphalt and asphalt mixtures over time, two main periods can be distinguished. Up to a certain point, asphalt aging processes can be characterized only as processes of strengthening the structure; the adhesion of asphalt to the surface of mineral grains improves (the acid number of asphalt increases) and it interacts better with carbonate mineral materials, as a result of which the water resistance and frost resistance of asphalt mixtures increase. The increase in asphalt viscosity during this period leads to an optimal increase in the strength and deformation stability of asphalt mixtures. Then comes the period to which the term “bituminous aging” rightly refers: due to the increase in asphalt brittleness, the dynamic strength decreases, which leads to a decrease in the resistance of the pavement to traffic loads.

The slowing down of pavement aging can be achieved by using dense bituminous mixtures (with minimum porosity), as well as periodically renewed surface treatment of the pavement, which prevents the migration of oxygen to the asphalt layer. The decrease in air and water circulation in the asphalt pavement rapidly slows down the process of asphalt oxidation [27]. However, in all cases, the asphalt aging process leads to the loss of the functional properties of asphalt mixture, and the pavement must be milled and replaced. As a result of grinding, a mixture of partially aged asphalt and mineral filler is created (RAP). The task solved in this paper is to restore the functional properties of the asphalt binder contained in the composition of reclaimed asphalt by using special activating additives [28].

Restoration of asphalt properties is aimed at restoring its original characteristics, such as elasticity, viscosity, and adhesion to mineral aggregate. To restore the properties of the asphalt binder in the composition, it is possible to add regenerating activators or carry out non-chemical treatment associated with the cracking of high-molecular bitumen compounds. However, the process of passing through the cracking column requires ensuring high-temperature values and is energy-intensive. Moreover, this process is much more complicated due to the presence of mineral filler.

The most technologically effective is the process of treating old asphalt binder/mixture with activators, since there is no need to carry out energy-intensive heating of the mixture. In addition, the properties of the asphalt binder are restored due to physical and chemical processes. Given the fact that road asphalt belongs to the group of asphalts with a “sol–gel” structure, which under the influence of aging turns into a “gel” structure, the addition of an activator will allow for the restoration of the chemical composition of asphalt. The activator weakens the “gel” structure of mature bitumen and transforms part of its structure into a “sol”. As a result of the hydrogenation reaction, asphaltenes are exposed to hydrogen, which leads to the rupture of bonds between carbon atoms and the formation of lighter hydrocarbons. For these purposes, it is proposed to use active plasticizers containing oils and resins. A similar assumption was adopted in this research and the use of a dedicated chemical activator was proposed, allowing the use of reclaimed asphalt as a granular base for cold repairs.

Studies on the qualitative and quantitative selection of activating additives were carried out in several stages. In the first, a qualitative assessment of the reclaimed asphalt was made, with particular emphasis on the properties of the recovered binder. A preliminary assessment of the effect of activating additives on asphalt binder properties was made. In the next step, optimization analyses were carried out in the scope of the selection of the amount of additive. In order to maintain the best repeatability of the results, the tests were carried out on road bitumen 35/50 in three states: original (non-aged NA), after short-term aging (RTFOT), and after long-term aging (PAV). The basis was MSCR tests in a dynamic shear rheometer. The goal of optimal dosage of additives was achieved by approximating the test results, determining the susceptibility to permanent deformations and elastic recovery, and then referring them to the results obtained on binders in their original state or after RTFOT aging. The next stage was testing the developed mixtures in order to assess their mechanical properties, in particular tensile strength. This parameter will be the so-called decision criterion at the stage of determining the activator composition. The result of the study is the development of the optimal composition of the cold mixture for pothole repairs based on RAP, which was then applied as part of maintenance treatments on the selected road pavement.

## 2. Materials for Testing and Characterization of Prepared Additives

### 2.1. Reclaimed Asphalt

**Figure 1:**
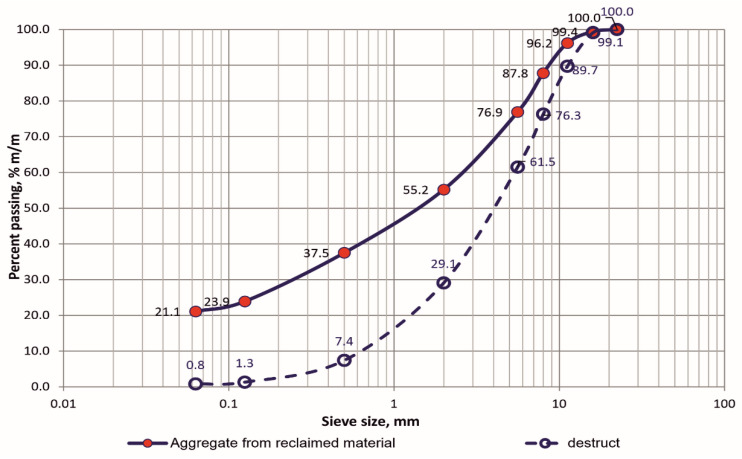
Grading curves of RAP and the mineral mixture after extraction.

Reclaimed asphalt marked as RAP was obtained by milling the surface of a municipal road in the vicinity of Warsaw. The grading of the RAP and the mineral mixture after extraction was determined in accordance with the assumptions of the PN-EN 12697-2:2019-08 standard [29]. The results of the grading of the reclaimed material and the mineral mixture from RAP are presented in Figure 1. The recovery of the mineral mixture and the determination of the soluble binder content in the reclaimed asphalt were carried out using the mass difference method in an automatic extractor according to PN-EN 12697-1:2020-08 [30]. The soluble asphalt content in RAP is 5.6%. No tar binder was found in the tested specimen. The petrographic evaluation of the mineral mixture obtained from the reclaimed material showed that it contains aggregates from rocks such as basalt, melaphyre, granite, and greywacke.

### 2.2. Selection of Activating Additives

Road bitumen, characterized by a “sol–gel” structure, undergoes a transition to a “gel” structure during aging due to chemical and molecular changes. Application of a chemical activator facilitates positive modifications in the group chemical composition of bitumen, resulting in a reduction in asphaltene content and an increase in oil fractions. The activator weakens the “gel” structure of aged bitumen, transforming it into a “sol” structure. The activator employed consists of alkyl phenates, alkyl naphthenes, high-molecular-weight aromatic hydrocarbons, petroleum distillates, hydrogenated olefin oligomers, and esters. It was considered that asphaltenes can be reduced to oils through hydrogenation, a process involving exposure to hydrogen, which induces the cleavage of carbon–carbon bonds, forming lighter hydrocarbons such as oils. This process enhances the plastic properties of bitumen and improves its resistance to fatigue deformations. The combined effects of these processes underpin the research. At this stage, the use of bio-oils or similar materials was not considered [31,32]. The primary objective is to develop a composition for restoring the functional properties of road surfaces across the widest possible temperature range using asphalt granulate in cold technology. Future investigations will explore the potential incorporation of bio-oils and reagents derived from “green technologies”.

The first additive investigated in this paper (W1) is based on the use of a mixture of oxidized distillate with petroleum esters, a mixture of aromatic hydrocarbons with petroleum resins, cellulose rubber powder, with the addition of epoxidized soy resin. The second additive (W2) is created using the BRW IV PPS bitumen additive in a mixture with class R separation oil, a mixture of aromatic hydrocarbons and petroleum resins. The general characteristics of the additive properties are presented in Table 1. Additive W2 is characterized by higher viscosity. It is worth noting that the patent procedure for the additives presented in this paper has been initiated, and for this reason, no detailed information on their composition has been provided.

## 3. Methodology for Optimizing Additive Content Using the MSCR Test

The first and most important problem in the case of preparing mixtures of binders and additives is dosage. Therefore, at the beginning, an analysis was carried out to determine the appropriate dose of the additive so that it would be possible to restore the compliance of the aged binder to the original state (before PAV aging). For this purpose, three different percentage values of the W1 and W2 additive were adopted (see Table 2). The dosage marked as d1 results from the mass analysis carried out on the basis of the dosage determined in the preliminary tests. It is worth noting that the consistency of the aged 35/50 bitumen with W1 and W2 additives in the dosage marked as d1 (i.e., 34.72% and 45.09%, respectively) was almost liquid. Therefore, the next two dosage levels were assumed as d2 = d1/2 and d3 = d2/2, obtaining consistency closer to a solid/viscoelastic liquid. The three selected dosages are designed to encompass a range of mixture consistencies, from liquid to nearly solid. There is no strict requirement to employ three dosages, as additional reductions can be implemented to obtain fourth and fifth data points for approximation. This approach is thus considered versatile and broadly applicable.

For the study, 35/50 unmodified binder was selected, which was successively aged by RTFOT and PAV [33,34], and then additives were added to the binder. The MSCR tests according to ASTM D7405 [35,36] were performed to assess the rheological properties the composed binders [35,37,38,39] carried. This test consists of repeated loading and unloading of a sheared sample with a stress pulse with a maximum shear stress value of 0.1 kPa and then 3.2 kPa [40,41] at a temperature of 60 [°C]. Based on the conducted tests, the parameters R_0.1kPa and R_3.2kPa are determined, which represent elastic shearing recovery and are expressed in %. Obtaining a high value of shearing recovery means that the material is more elastic than plastic (in the case of the value equal to 100%, the material is fully elastic, while in the case of a low percentage recovery, it is becoming more plastic, which translates into substantial permanent deformation in the pavement). Additionally, it indicates so-called compliance to irreversible creep deformation Jnr_0.1kPa and Jnr_3.2kPa (the higher the compliance, the greater the sensitivity to permanent deformation, which consequently translates into rutting of the mineral–asphalt mixture). Summarizing, obtaining a high value of recovery and a low value of compliance is the goal of this analysis. The MSCR test is a test that is intended to allow for a rational assessment of polymer-binders or other binders when subjected to various types of modifications.

### 3.1. MSCR Test Results

**Figure 2:**
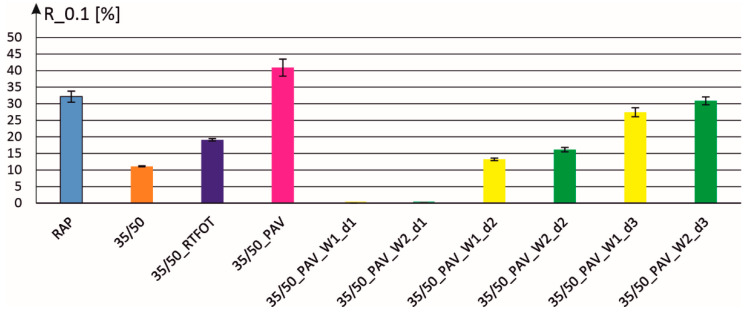
Elastic recovery parameter R_0.1 for individual binders with two additives and at three dosage levels.

**Figure 3:**
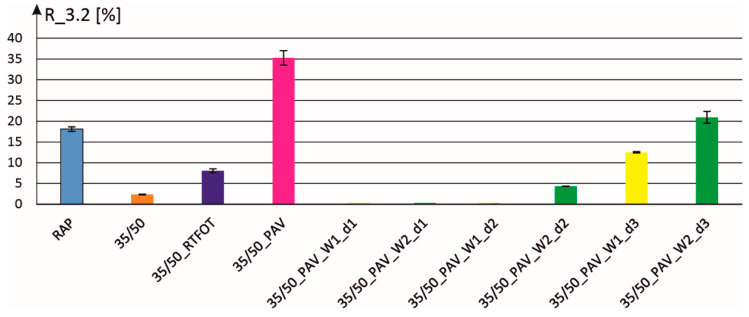
Elastic recovery parameter R_3.2 for individual binders with two additives and three dosing levels.

**Figure 4:**
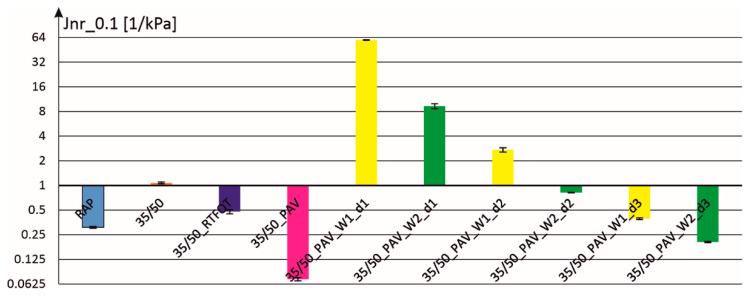
The parameter of compliance to permanent deformation at a load of 0.1 [kPa] for individual binders with two additives and at three dosage levels.

**Figure 5:**
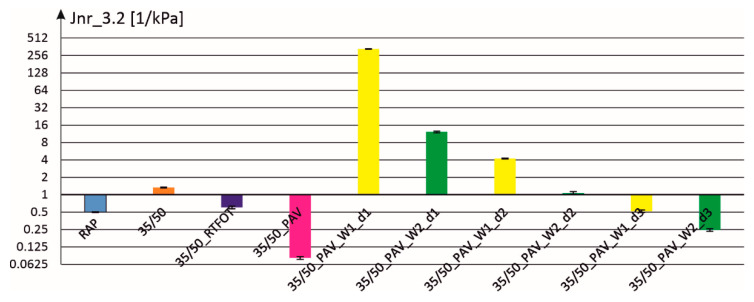
The parameter of compliance to permanent deformation at a load of 3.2 [kPa] for individual binders with two additives and at three dosage levels.

The results of MSCR rheological tests are presented in Figure 2, Figure 3, Figure 4 and Figure 5. Analyzing, for example, the parameter R_0.1 (elastic shearing recovery in percent at a lower level of stress excitation), the obtained results are related to those obtained for 35/50 binder in its original state, after RTFOT aging and after RTFOT + PAV aging (marked as PAV in the figure). Additionally, this parameter was also determined for bitumen recovered from RAP, which is the basis for cold mixes created for pothole repairs. Next, Figure 2 presents the results for binders created by mixing aged 35/50 bitumen (RTFOT + PAV) and additives W1 and W2 at three dosing levels d1, d2, and d3 (see Table 2). It can be seen that the parameter R_0.1 takes on a zero value at dosing level d1, and reducing the content of additives in the binder results in an increase in this parameter.

In the case of the parameter R_3.2 (Figure 3), zero values of elastic recovery for the additive dosage d1 are also obtained. Moreover, in the case of the additive W1, with the dosage d2 no elastic recovery is achieved. Non-zero values of the parameter R_3.2 were obtained only for the smallest mass content of the additive, i.e., d3, for both additives and for the dosage d2 for the additive W2. The parameter of elastic recovery at a high-stress level R_3.2 is usually used to assess modified and highly modified binders. In the case under consideration, paving grade bitumen is involved, rendering the MSCR test significance lower in the assessment of rheological properties.

The following Figure 4 and Figure 5 show the parameters of compliance to permanent deformations at the load levels of 0.1 and 3.2 [kPa]. In both cases, the compliance value for binder–additive mixtures with dosing level d1 was so high that, for better readability of the figures, the scale on the ordinate axis was changed to a logarithmic one (with base equal to 2).

### 3.2. Proposal of a Method for Determining Dosage Based on Compliance to Permanent Deformations and Elastic Recovery MSCR Parameters

Analyzing the results presented in Section 3.1, a method for determining the dosage was proposed that allows obtaining a binder–additive mixture with equivalent elastic recovery or equivalent compliance to permanent deformations.

**Figure 6:**
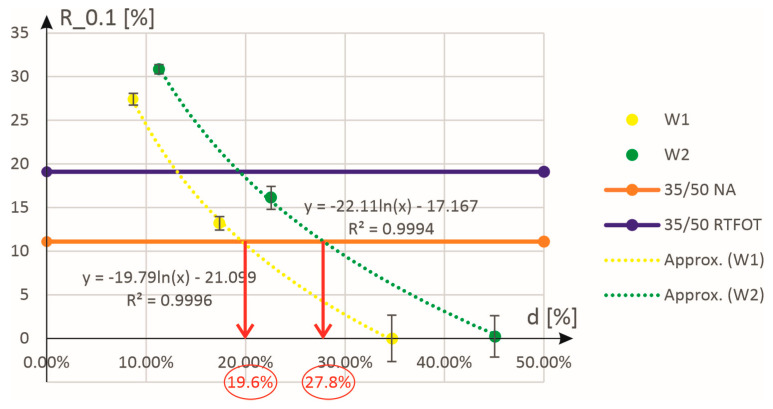
Approximation of experimental results for the elastic recovery R_0.1 as a function of the mass content (expressed in %) of the additives W1 and W2.

In the case of the parameter R_0.1, the elastic recovery as a function of the additive dosage (W1 and W2, see Figure 6) was plotted on the graph. These are the filled circular markers visible on the graph, through which approximations were then performed with functions of the following form:

(1) $$R\_0.1\left(d\right)=a\cdot \ln\left(d\right)+b,$$

where d is the activator dosage (in%) while a and b are parameters determined in regression analysis. As part of the approximation, the parameters a and b were determined to minimize the mean square error. As shown in Figure 6, the proposed function ideally fits the experimental results, as evidenced by the determination coefficients at the level of 0.99. Additionally, in this figure, the horizontal lines determine the value of elastic recovery at a low level of stress excitation obtained for the original 35/50 binder (not subjected to aging and RTFOT-aged). Therefore, the intersection points of the approximation functions and the horizontal lines determine the dosage that allows obtaining elastic recovery for the unaged or RTFOT-aged asphalt binders. For example, Figure 6 illustrates (red arrows) how to determine the dosage that achieves an elastic recovery R_0.1 equivalent to that of the unaged 35/50 binder. The results highlighted in red font can be found in the sixth column of Table 3.

**Figure 7:**
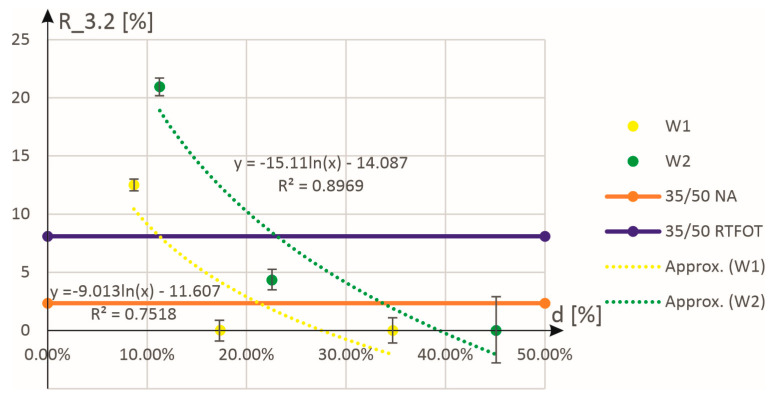
Approximation of experimental results for elastic recovery R_3.2 as a function of the mass content (expressed in %) of the additives W1 and W2.

Figure 7 shows an approximation of the experimental results for elastic recovery at high-stress levels. It can be seen that this is not as good an approximation as the data for the lower stress level. In this case, the values of the coefficient of determination were obtained as equal to 0.75 for the W1 additive and 0.90 for the W2 additive.

**Figure 8:**
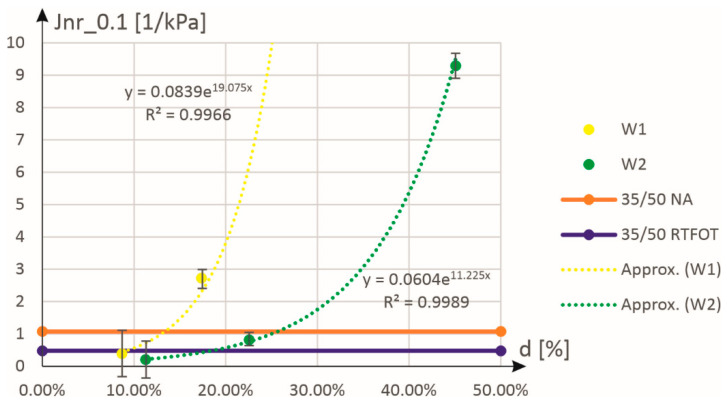
Approximation of experimental results on compliance to permanent deformations Jnr_0.1 as a function of the mass content (expressed in %) of the W1 and W2 additives.

**Figure 9:**
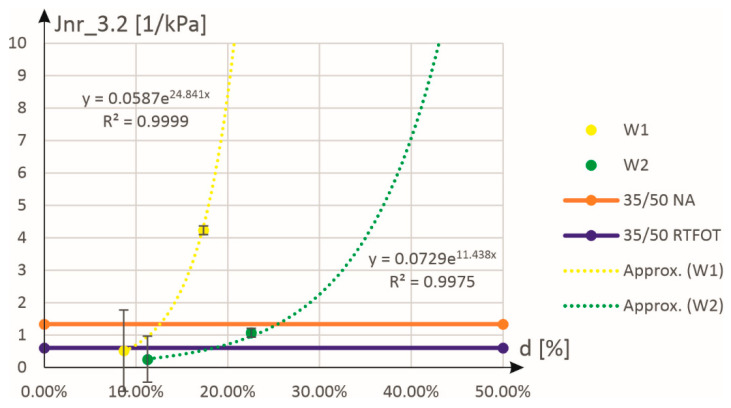
Approximation of experimental results on compliance to permanent deformations of Jnr_3.2 as a function of the mass content (expressed in %) of the W1 and W2 additives.

Figure 8 shows the approximation of the results for compliance to permanent deformations. In this case, the approximation function was assumed in the form of an exponential function:

(2) $$\mathrm{Jnr}\_0.1\left(d\right)=a\cdot \exp\left(b\cdot d\right)$$

where the interpretation of equation symbols is as for Equation (1). As can be seen in Figure 8, this function perfectly approximates the experimental results (determination coefficients at the level of 0.99) for both stress excitation levels (see also the graphs in Figure 9).

**Figure 10:**
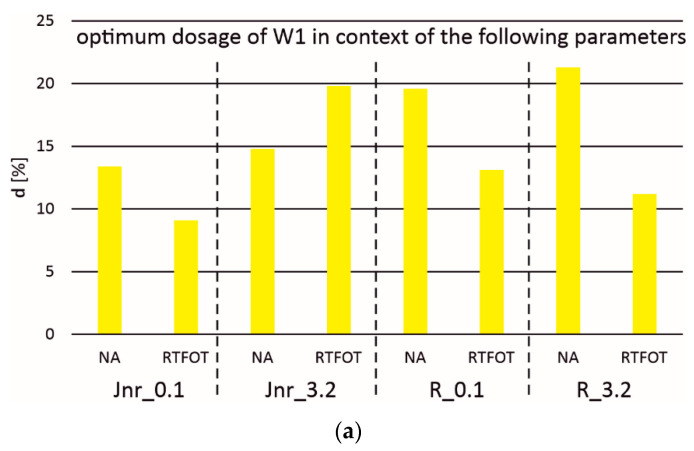

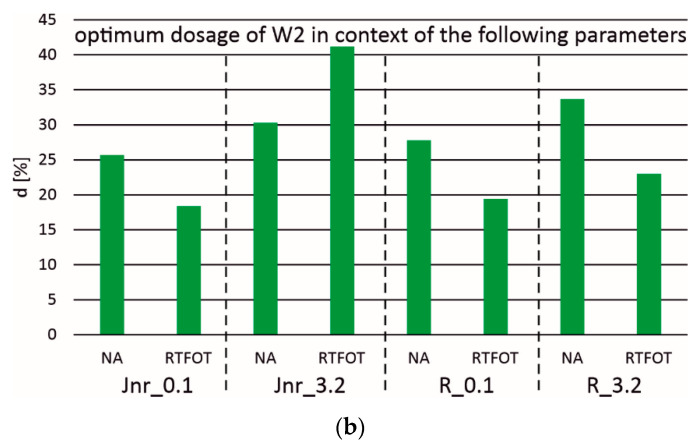
Optimum dosage of additives W1 (**a**) and W2 (**b**) in the context of the following MSCR parameters, taking into account aging.

Table 3 and Figure 10 present the mass content values of activators W1 and W2 relative to the aged binder 35/50 (RTFOT + PAV). These values were determined based on the equivalence of compliance to permanent deformations (Jnr_0.1 or Jnr_3.2) and elastic recovery (R_0.1 or R_3.2) with those of the non-aged or RTFOT-aged asphalt 35/50. Analysis of the results indicates that the lowest percentage content was achieved when assuming equivalence of compliance to Jnr_0.1 compared to the asphalt after technological aging, with values of 9.1% for activator W1 and 18.4% for activator W2. The requirement to use nearly twice as much activator W2 compared to W1 stems not only from differences in their chemical composition but also from variations in their consistency.

Table 4 presents the parameters of the approximation of experimental results using Equations (1) and (2). The coefficients of determination for these approximations, ranging from 0.75 to 0.99, are shown in Figure 6, Figure 7, Figure 8 and Figure 9. For further analyses, the mass share values were considered reliable when determined based on the equivalence of compliance to permanent deformations (Jnr_0.1) with the results obtained for the binder after technological aging. These values are highlighted in gray cells in Table 3 and Table 4.

### 3.3. The Influence of Activating Additives on Asphalt Recovered from Reclaimed Material

After determining the optimal content of chemical activators W1 and W2 in Section 3.2, here the effect of activators on binders recovered from RAP is analyzed. The results of basic tests carried out on this binder are presented in Table 5, which were the basis for assessing the effectiveness of the used additives W1 and W2. Asphalt mixtures with additives were prepared using the so-called optimal dosage d0 (see Section 3.2).

Adding both W1 and W2 activators has a clear effect on the change in the consistency of the old binder. It significantly increases penetration at 25 °C, lowers the softening temperature, favorably lowers the breaking temperature, and improves the elastic recovery. Activator W1 is distinguished by significantly greater efficiency, where positive changes in the properties of old asphalt binder can be achieved using half the activator content (compared to W2).

**Figure 11:**
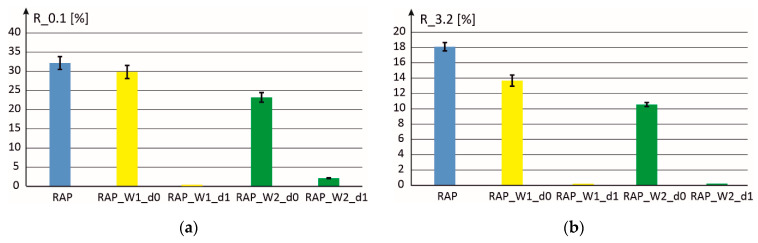
Elastic recovery parameters: (**a**) R_0.1kPa and (**b**) R_3.2kPa, for mixtures with two additives (W1 and W2) and with two volume fractions d0 (optimal value) and d1.

**Figure 12:**
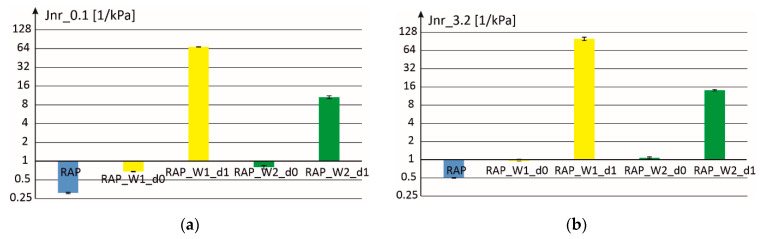
Parameters of compliance to permanent deformations: (**a**) Jnr_0.1 and (**b**) Jnr_3.2, for mixtures with two additives (W1 and W2) and with two volume fractions d0 (optimal value) and d1.

Figure 11 and Figure 12 present the results obtained for two doses of activators W1 and W2 (d0 and d1). It can be seen that in the case of dosing level d1 the consistency of the obtained mixture is almost liquid, which means significant compliance to permanent deformations and a minimum value of the so-called elastic recovery. In the case of dosing level d0 of the W1 and W2 additives, it was possible to maintain almost the same values of elastic recovery parameters as for the binder extracted from RAP, while significantly increasing the compliance to permanent deformation.

In Figure 12, the scale on the vertical axis was changed into exponential (with base equal to 2) to properly show the obtained results. In the case of dosages d1 the compliance to permanent deformation is very high while in the case of the binder recovered from RAP a very low value is obtained.

## 4. Indirect Tensile Strength Tests on Samples with Optimal Content of Chemical Activators W1 and W2

For further studies in the field of indirect tensile strength [45], tests were carried out on mineral–asphalt mixtures containing W1 and W2 additives, as shown in Table 3. Various sample conditioning procedures in terms of temperature and curing time were taken into account. Studies were carried out to investigate the effect of time and conditions of “maturing” the mixture after its production using activators W1 and W2. For this purpose, two samples of the repair mixture weighing 60 kg each were prepared, the first using composition W1 and the second using composition W2.

The ingredients (RAP and additives) were mixed under laboratory conditions in a typical laboratory mixer designed for mineral–asphalt mixtures at a temperature of 20 °C for 10 min. Then, the obtained mixture was unloaded from the mixing device and packed in a tightly closed container for “maturing” at 20 °C. After 7 days, samples of the resulting mixture were prepared and compacted using a Marshall compactor (2 × 35 hammer blows). In addition, half of the samples were stored at 0° and the other half at 20°. The cylindrical samples were subjected to a compression test along the generatrix, which realizes tensile splitting at intervals of 1, 2, 3, 7, 14, and 28 days after compaction. At the time of 24 h before the test, the samples that were stored in laboratory conditions were placed in a climatic chamber for conditioning at 0 [°C]. The mean values and standard deviation (95% confidence level) of average contact stress (ACS) and indirect tensile strength (ITS) are presented in Table 6.

An increase in strength is observed during the sample conditioning process. In the first two days, there is a slight decrease in strength indices, most likely associated with the diffusion of additives into the binder structure and deposition in the intercrystalline space. A further increase in tensile strength is most likely associated with the formation of a new spatial skeleton and the growth of structural connections in the mixture. A low temperature slows down the process of increasing tensile strength but does not stop it. In each case, there is a steady increase in tensile strength. The best results were achieved with the use of additive W2. However, the use of additive W1 also brought positive results. Both compositions are recommended for testing in real conditions.

## 5. Summary and Conclusions

As a result of the research carried out, a basic composition of a cold repair mix with activators W1 and W2 was obtained. Studies were carried out on the effect of various additives on the physical and mechanical properties of the final repair mix made of reclaimed asphalt. The compositions and technology for obtaining an organic–mineral mixture were developed using the new and complex additives W1 and W2. A trial batch of the repair mixture was produced and mechanical tests were carried out.

### 5.1. Conclusions Based on Binder Tests

- For each of the analyzed additives (i.e., W1 and W2) there is an optimal mass content in relation to the binder, which allows for obtaining the best values of basic features (penetration, Fraass breaking point, etc.) and rheological properties determined in the MSCR test;
- The consistency of the W2 additive is much more viscous than that of the W1 additive, which significantly affects the obtained results, i.e., at the dosage marked as d1, it can be assumed that the 35/50 aged binder mixtures with the W1 additive are liquids, so the MSCR test is not adequate in this case;
- Activators W1 and W2 do not affect binders in a way analogous to classical modifications with SBS copolymer. MSCR results obtained for mixtures at different mass content levels place them below the curve separating typical modified and unmodified asphalt binders;
- Physical aging (time elapsed since the mixtures were made) has a positive or negative effect on MSCR parameters, depending on the dosage. This conclusion allows us to formulate the observation that there is an optimal dosage level for each additive;
- Additives W1 and W2 have shown their usefulness in activating the binder recovered from RAP. This is manifested by increased compliance to permanent deformations while maintaining a high value of elastic recovery at a low-stress level. It is worth emphasizing, however, that excessive amounts of additives reverse this tendency;
- Adding both W1 and W2 activators has a clear effect on the change in the consistency of binder recovered from RAP. It significantly increases penetration at 25 °C, lowers the softening temperature, favorably lowers the breaking temperature, and improves the elastic recovery. Activator W1 is distinguished by significantly greater efficiency, where positive changes in the properties of the old binder can be obtained using half the activator content. These changes are clearly more beneficial, because, at a comparable softening temperature and significantly higher penetration at 25 °C, better results were obtained in terms of compliance to permanent deformations and elastic recovery.

### 5.2. Conclusions Based on Indirect Tensile Strength Tests of Repair Mixtures

- With optimal doses of activators W1 and W2, very high values of average contact stresses were obtained, exceeding 3 [MPa] in almost all cases (in the best variants for the matured mixture even 6 [MPa]). These values present indirect tensile strength values equal to 0.24 [MPa] and 0.48 [MPa], respectively;
- The influence of the maturation time on the obtained strengths was observed. During the first two days, the indirect tensile strength decreases slightly and then increases significantly in the following days (tests were performed after 1, 2, 3, 7, 14, and 28 days);
- For a selected group of samples prepared in the Marshall apparatus, a study of the regenerative capabilities of the analyzed repair mixtures was conducted. Namely, after performing the indirect tensile strength test on the material of the split samples, further samples were prepared and the tests were carried out again, obtaining results at a comparable level. This means that the proposed mixture has the ability to self-repair after the occurrence of cracks (these results were not presented above);
- The water and frost resistance test was conducted in a standard manner, obtaining ITSR of 51.1% and on samples conditioned for 28 days (ITSR is 56.2%). Analyzing these results in relation to typical “hot” mixtures, it can be seen that freezing cycles significantly reduce the tensile strength of the tested mixture for pothole repairs. This conclusion is based only on initial test results and has to be justified in a wider testing campaign.

### 5.3. Conclusions Based on Preliminary Application in Real Conditions

**Figure 13:**
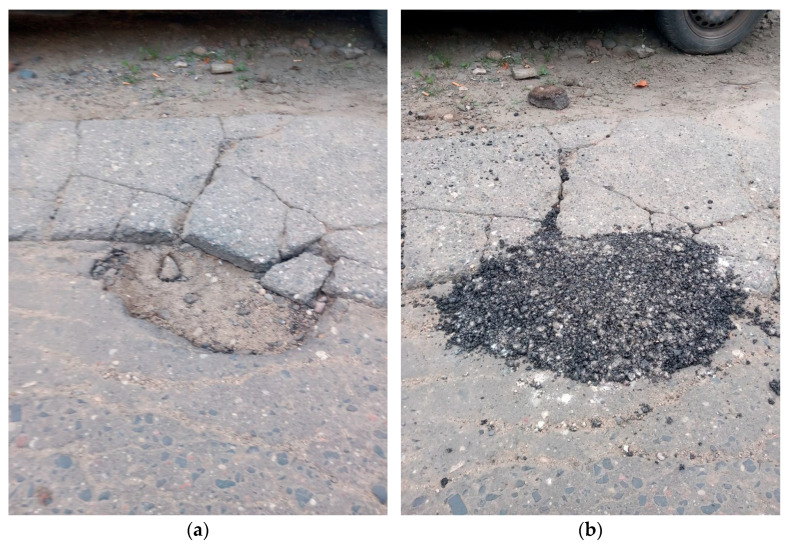
The exemplary application of the mixture based on activator W2: (**a**) before repair, (**b**) just after filling the pothole.

This article presents the initial applications of a pothole repair agent on low-category residential roads (Figure 13). The objective was to conduct preliminary repairs during the winter period without significant delays, even if performed in a partially informal manner. The repairs were carried out under challenging conditions: in one instance following heavy rainfall, and in another during light snowfall, using only basic tools. The success of these interventions is evidenced by the fact that, after 60 days, the filled potholes remain intact, with no observed cracks or rutting. However, it is anticipated that the proposed solutions may face critical challenges during the summer period when pavement temperatures exceed 60 °C. Should the patent process prove successful, more systematic studies on an actual road are planned. These studies will involve proper preparation of pothole edges, repairs conducted under varying weather conditions, and systematic monitoring of the repaired potholes, traffic, and weather. The outcomes of these studies will be the subject of a subsequent publication.

### 5.4. Discussion of Future Work

The article presents preliminary results regarding the application of chemical activators to transform reclaimed asphalt pavement (RAP) into a fully functional material suitable for year-round pothole repairs of road surfaces. It addresses the issue of determining the optimal proportions of activators relative to RAP based on the rheological properties test of asphalt binders (MSCR). The study was extended to the final product, analyzing its mechanical properties (ITS and ACS) as a function of temperature and time, as well as conducting pothole repairs under relatively adverse weather conditions (rain and snow). However, it should be noted that further research is necessary. Among the most critical directions for future investigation are the following:

- Functional comparative studies of the proposed pothole repair mixture against commercially used materials;
- Expansion of testing the properties of the obtained product, including rutting resistance, surface abrasion (DSD), fracture resistance (SCB), and resistance to water and frost action (ITSR);
- To enhance the interpretation of the obtained results, it is planned to integrate relevant concepts from structural integrity monitoring and hybrid modeling techniques. Fuzzy similarity can be employed in the context of this study to evaluate the performance of modified binders and recycled mixtures in a more flexible and continuous manner, accounting for variability in experimental data and operating conditions. This approach enables the comparison of similar mechanical properties despite numerical differences, the classification of performance without rigid thresholds, and the analysis of mixture stability under diverse environmental conditions [46];
- Carrying out extensive field tests of the proposed mixtures. These studies will involve proper preparation of pothole edges, repairs conducted under varying weather conditions, and systematic monitoring of the repaired potholes, traffic, and weather.

## Figures and Tables

**Table 1:** Properties of W1 and W2 additives (activators).

Properties	W1	W2
Water solubility	Insoluble	Insoluble
Solubility in organic solvents	Soluble	Soluble
Appearance	Oily liquid	Oily liquid
Density [g/cm^3^]	0.95–0.98	0.95–1.03
Dynamic viscosity at temperature 20 [°C]	19–23	31–37

**Table 2:** The amount of additive in relation to the binder (m/m).

	Dosage	d1	d2	d3
Additive				
W1		34.72%	17.36%	8.68%
W2		45.09%	22.55%	11.28%

**Table 3:** Determination of equivalent values of the mass content of additives W1 and W2 depending on the given parameter (obtained values are optimal dosages d0 for different assumptions).

	Jnr_0.1		Jnr_3.2		R_0.1		R_3.2	
Condition	NA	RTFOT *	NA	RTFOT	NA	RTFOT	NA	RTFOT
W1	13.4	9.1	14.8	19.8	19.6	13.1	21.3	11.2
W2	25.7	18.4	30.3	41.2	27.8	19.4	33.7	23.0

* gray background color indicates dosages used for further analysis.

**Table 4:** The parameters in Equations (1) and (2) determined in the optimization process.

	Jnr_0.1		Jnr_3.2		R_0.1		R_3.2	
Parameter	a *	b	a	b	a	b	a	b
W1	0.0839	19.075	0.0587	24.841	−19.79	−21.099	−9.013	−11.607
W2	0.0604	11.225	0.0729	11.438	−22.11	−17.167	−15.11	−14.087

* gray background color indicates parameters of Equation (2) used for further analysis.

**Table 5:** Test results of asphalt recovered from RAP.

Property	Type of Binder			Standard
	RAP	RAP/W1 (9.1%W1)	RAP/W2 (18.4%W2)	
Penetration at temperature 25 °C, total loading 100 g, loading time 5 s, in units 0.1 [mm]	20	55	40	PN-EN 1426 [42]
Softening point R&B [°C]	63.3	53.4	54	PN-EN 1427 [43]
Fraass breaking point [°C]	−9	−17	−11	PN-EN 12593 [44]

**Table 6:** Mean values and standard deviation (95% confidence level) of average contact stress (ACS) and indirect tensile strength (ITS) (cylindrical samples) at 0 and 20 [°C].

Mat. Time, [days]	W1 0 [°C] [MPa]		W1 20 [°C] [MPa]		W2 0 [°C] [MPa]		W2 20 [°C] [MPa]	
	ACS	ITS	ACS	ITS	ACS	ITS	ACS	ITS
1	3.14 ± 0.12	0.24 ± 0.01	3.14 ± 0.13	0.24 ± 0.01	4.39 ± 0.64	0.34 ± 0.05	4.39 ± 0.65	0.34 ± 0.05
2	3.32 ± 0.15	0.26 ± 0.01	3.40 ± 0.09	0.26 ± 0.01	3.86 ± 0.56	0.30 ± 0.04	3.91 ± 0.58	0.30 ± 0.05
3	2.96 ± 0.12	0.23 ± 0.01	3.10 ± 0.18	0.24 ± 0.01	4.35 ± 0.28	0.34 ± 0.02	4.51 ± 0.93	0.35 ± 0.07
7	3.44 ± 0.03	0.27 ± 0.02	3.52 ± 0.39	0.27 ± 0.03	4.34 ± 0.07	0.34 ± 0.01	4.98 ± 1.34	0.39 ± 0.10
14	3.59 ± 0.17	0.28 ± 0.01	3.95 ± 0.32	0.31 ± 0.02	4.44 ± 0.71	0.34 ± 0.06	5.32 ± 0.27	0.41 ± 0.02
28	3.37 ± 0.32	0.26 ± 0.01	3.68 ± 0.36	0.29 ± 0.02	5.47 ± 0.78	0.42 ± 0.06	6.19 ± 0.51	0.48 ± 0.04

## Data Availability

The original contributions presented in this study are included in the article. Further inquiries can be directed to the corresponding author.
